# Combination of Selected MET and EGFR Inhibitors Decreases Melanoma Cells’ Invasive Abilities

**DOI:** 10.3389/fphar.2019.01116

**Published:** 2019-10-01

**Authors:** Aleksandra Simiczyjew, Katarzyna Pietraszek-Gremplewicz, Ewelina Dratkiewicz, Marta Podgórska, Rafał Matkowski, Marcin Ziętek, Dorota Nowak

**Affiliations:** ^1^Department of Cell Pathology, Faculty of Biotechnology, University of Wroclaw, Wroclaw, Poland; ^2^Department of Oncology and Division of Surgical Oncology, Wroclaw Medical University, Wroclaw, Poland; ^3^Lower Silesian Oncology Center, Wroclaw, Poland

**Keywords:** gefitinib, lapatinib, foretinib, melanoma, invasion, invadopodia, EGFR, MET

## Abstract

We have previously shown that combination of foretinib, an inhibitor of MET (hepatocyte growth factor receptor), with gefitinib or lapatinib, inhibitors of EGFR (epidermal growth factor receptor), has a synergistic cytotoxic effect on melanoma cells. However, there are cancer cells resistant to drugs’ treatment which are still able to invade. Thus, in this study, we examined the influence of these drugs on invasive abilities of melanoma cells. To investigate cell migration and invasion, Transwell inserts and wound healing assay were used. Cell viability was evaluated by XTT method, while invadopodia formation by immunocytochemistry. Level of phosphorylated Src kinase (pSrc) was verified by Western blot. Proteolytic activity of cells was analyzed using gelatin conjugated with fluorescein degradation assay and gelatin zymography. Combination of used inhibitors diminished cell movement, resulting in smaller distances covered by cells, and decreased the ratio of cells with ability to cross the Transwell inserts. These inhibitors induced changes in formation of invadopodia and actin cytoskeleton organization. Their application also decreased the level of pSrc kinase. Furthermore, used drugs led to reduction of proteolytic activity of examined cells. Our data support the idea that simultaneous targeting of EGFR and MET could be a promising therapeutic strategy inhibiting not only tumor cell growth but also its metastasis.

## Introduction

In the recent years, more specific, targeted therapies against melanoma have appeared, mostly directed against BRAF kinase. Unfortunately, persistence of BRAF inhibitors’ effect on cells is limited due to the emerging cell resistance to this treatment, connected to reactivation of the MAPK (mitogen-activated protein kinase) pathway (Flaherty et al., 2012). To address this problem, MEK inhibitors were recently introduced to melanoma therapy. Combined BRAF and MEK blocking, in comparison to the single-agent BRAF inhibition, delayed the appearance of the resistance but was not able to completely abolish its development. Various mechanisms leading to resistance against BRAF inhibitors have been identified, including the one involving EGFR (epidermal growth factor receptor). Overexpression of EGFR often occurs in advanced stage of melanoma (Kovacs et al., 2015). It was shown that ectopic expression of EGFR in melanoma cells was sufficient to cause vemurafenib (BRAF inhibitor) resistance (Gross et al., 2014). To solve this problem, EGFR inhibitors can be employed. MET (hepatocyte growth factor receptor) was also demonstrated to be connected with malignant skin cancer development, and the level of its expression seems to be related to the stage of malignancy in melanoma (Lee et al., 2011). Due to the involvement of EGFR and MET in melanoma progression, these receptors can be promising therapeutic targets.

EGFR is often overexpressed in human cancer cells, which correlates with tumor progression and worse prognosis for patients (Boone et al., 2011). After ligand binding, EGFR receptor undergoes dimerization, which then leads to its autophosphorylation on tyrosine residues and activation of various signaling pathways, including the most significant PI3K (phosphoinositide 3-kinase)/AKT (protein kinase B) and MAPK. These cascades of signal transduction participate in regulation of cell proliferation, prevent apoptosis and promote cell invasion (Di Domenico and Giordano, 2017). Therefore, *EGFR* gene amplification is associated with higher cancer invasion capacity and formation of metastasis (Rákosy et al., 2007). Additionally, cancer cell migration connected with epithelial-mesenchymal transition is enhanced by activation of EGFR. Blocking of this receptor by inhibitors or antibodies decreases the ability of cancer cells to invade (Al Moustafa et al., 2012). The PIK3/AKT pathway is also essential for metastasis of esophageal squamous cell carcinoma, since its inhibition reduced motility of cancer cells (Li et al., 2017).

Higher level of MET is also frequently reported in several types of cancer, such as lung, breast, and colon cancers (Sierra and Tsao, 2011). Its autophosphorylation after ligand binding activates MAPK, STAT (signal transducer and activator of transcription protein family), and PI3K/AKT signal transduction pathways, which supports cancer cell survival, proliferation, and motility (Surriga et al., 2013). High level of MET also correlates with poor prognosis for patients, as a result of increased tumor growth and invasion (Sierra and Tsao, 2011), while higher expression of this receptor in primary uveal melanoma is associated with increased risk of liver metastasis (Surriga et al., 2013).

Stimulation with EGF, a major chemoattractant for invading cancer cells, results in activation of EGFR downstream signaling pathways. This leads to generation of protrusive force that enables cancer cells to form invadopodia, penetrate through the ECM, and form metastasis (Mader et al., 2011). These actin-rich adhesive structures secrete proteases digesting elements of extracellular matrix (ECM), thus forming the path used by cancer cells to migrate through surrounding microenvironment (Yamaguchi, 2012). MET may also localize to invadopodia along with cortactin, one of the main migratory protrusion component, and promote phosphorylation of this protein (Rajadurai et al., 2012). It was shown that both EGFR and MET signaling regulate invadopodia formation, and ECM degradation (Mader et al., 2011; Rajadurai et al., 2012).

Due to the involvement of EGFR and MET signaling in regulation of cell invasion, agents blocking their activity could be used as anti-metastatic drugs. However, independently used inhibitors require application of higher concentrations and more rapidly lead to the occurrence of resistance to this type of agents (Lovly and Shaw, 2014). Additionally, single-agent therapy may not be effective due to the expression of both receptors in cancer cells. Another reason is the crosstalk between the downstream signaling cascades, which can cause the therapeutic resistance to EGFR or MET inhibitors used as a monotherapy (Easty et al., 2011). For this reason, it is likely that dual inhibition of MET and EGFR is required to reduce the motility of cells.

Here, we focused on the influence of simultaneous treatment of melanoma cells with selected inhibitors of EGFR - gefitinib or lapatinib, and MET - foretinib. In our previous work, we showed that combination of these drugs results in a synergistic cytotoxic effect on the viability and proliferation of melanoma cells derived from primary tumor, and metastasis. These mixtures of inhibitors also decreased AKT and ERK phosphorylation and led to the appearance of polyploidal cells, and massive enrichment in the G2/M phase. Additionally, after treatment with pairs of foretinib/lapatinib or foretinib/gefitinib, cells exhibited increase in size with more distinct stress fibers and unusually shaped nuclei. Combination treatment was much more effective against melanoma cells in tested parameters compared to the single-targeted approach (Dratkiewicz et al., 2018). Therefore, the aim of our study was to verify how combination of lapatinib or gefitinib with foretinib influences the invasion and migration of examined, primary and metastatic, melanoma cells.

## Materials and Methods

### Chemicals

Rabbit polyclonal anti-cortactin, mouse anti-phosphorylated Src, and mouse anti-GAPDH protein (glyceraldehyde 3-phosphate dehydrogenase) antibodies were purchased from Santa Cruz Biotechnology. Mouse anti-Src antibodies were obtained from Merck Milipore. Alexa Fluor 568–conjugated phalloidin, secondary anti-rabbit antibodies conjugated with Alexa Fluor 488, gelatin conjugated with fluorescein (FITC), fetal bovine serum (FBS), trypsin, glutamine, and penicillin/streptomycin/amphotericin B solution were obtained from Invitrogen, while DMEM from IITD PAN, Wroclaw. Dako Fluorescent Mounting Medium was purchased from Dako. EGF and Matrigel were obtained from BD Biosciences, while HGF from Sigma. Foretinib was purchased from Santa Cruz Biotechnologies; lapatinib and gefitinib from Selleckchem. Goat anti-mouse antibodies conjugated with horseradish peroxidase were obtained from Cell Signaling Technologies. All other chemicals were classified as analytical grade reagents.

### Cell Culture

The human melanoma cell lines derived from primary tumor—A375, and from metastasis—Hs294T were purchased from the American Type Culture Collection (ATCC), while WM9 cell line (derived from metastatic tumor) was purchased from Rockland Immunochemicals, Inc. Cells were grown in DMEM medium with 4.5 g/l glucose and 1.5 g/l NaHCO_3_ containing 10% FBS, 2 mM glutamine, and antibiotics (10,000 U/ml penicillin, 10 mg/ml streptomycin, 25 µg/ml amphotericin B). Cells were cultured in 25-cm^2^ tissue culture flasks (Sarstedt) at 37°C in 5%CO_2_/95% humidified air and passaged twice a week using 0.25% trypsin/0.05% EDTA solution (IITD PAN, Wrocław, Poland).

### Cytotoxicity Evaluation

Cell Proliferation Kit II (XTT) (Roche), a colorimetric assay used to assess cell number based on their metabolic activity, was used according to the manufacturer’s protocol. Cells were seeded in 96-well plates on top of thin layer of Matrigel (1 mg/ml). To obtain the coating of Matrigel, the plate was incubated for 30 min at 37°C and 5% CO_2_. Next, cells were covered with an additional Matrigel layer (and incubated for 1 h at 37°C), and then DMEM growth medium containing inhibitors was added on top of 3D Matrigel matrices. The XTT (2,3-bis-(2-methoxy-4-nitro-5-sulfophenyl)-2H-tetrazolium-5-carboxanilide)-labeling mixture was added after 24 h of cell growth in the presence of foretinib, gefitinib, lapatinib, or gefitinib/foretinib or foretinib/lapatinib in three-dimensional conditions. Absorbance was measured at 410 nm 3 h after XTT addition, and obtained values were background corrected. The mean cell viability was expressed as decrease in percentage of viability (absorbance) *vs*. control, non-treated cells at given time point (100% of viability). All conditions were performed in four replicates, and for each cell line, three independent experiments were conducted. Exact protocol of seeding cells as well as execution of test and cytotoxicity rate calculation was earlier described by Huyck et al. (2012).

### 2D and 3D Scratch Assays

Cells were seeded in ImageLock 96-well plates (Essen Bioscience) on the top of thin layer of Matrigel (1 mg/ml). To obtain the coating of Matrigel, the plate was incubated for 30 min at 37°C and 5% CO_2_. After 24 h, when the cells reached confluency, standardized wounds were created in all wells simultaneously using Wound Maker™ (Essen Bioscience). In the case of invasion assay, the cells and the cell-free zone were covered with an additional Matrigel layer. Then, DMEM growth medium containing inhibitors was added on the cell layer directly (migration assay) or on the top of 3D Matrigel matrices (invasion assay). Phase-contrast time-lapse images were captured using IncuCyte^®^ Live-Cell Analysis System with a time interval of 2 h using a 10x objective. Control cells and cells treated with inhibitors were allowed to invade the wound for 36 or 60h. The IncuCyte^®^ Scratch Wound Cell Migration Software Module was used for data analysis. The relative wound density was based on the increase in the area covered by the cells in time. The experiments were performed in triplicate, each condition consisting of four replicates.

### Migration Distances and Cell Trajectories

Cells were seeded in Matrigel-coated 96-well ImageLock plates, and 24 h later, growth medium containing inhibitors was added. Phase-contrast time-lapse photos were captured using IncuCyte^®^ Live-Cell Analysis System with a time interval of 2 h using a 10x objective. Control cells and cells treated with inhibitors were allowed to migrate for 48 h. The experiments were performed three times, and in each time, 40 cells were analyzed. An IncuCyte^®^ Scratch Wound Cell Migration Software Module and ImageJ software with Manual Tracking plugin (Schneider et al., 2012) were used for analysis.

### Transwell Invasion Assay

Cell invasion tests were performed using Transwell filters (BD Biosciences) placed in a 24-well plates. Before the experiment, cells were starved for 16 h in serum-free DMEM medium. Cells were seeded in medium without FBS in the absence (control) or presence of inhibitors onto Transwell filters coated with Matrigel (1 mg/ml). In the well medium containing 20% fetal bovine serum, 5 nM EGF and 30 ng/ml of HGF were present and served as a chemoattractant. After 24 h, non-invading cells and Matrigel on the upper side of the filters were removed. Cells which invaded through the membrane were fixed with 4% formaldehyde; nuclei were stained with Hoechst 33342 and counted under the fluorescent microscope. The results are presented as a relative invasion factor (%), and the number of control cells which invaded through the Transwell filters is set as 100%. The experiments were performed three times, and each independent experiment consisted of three measurements.

### Immunofluorescence

The subcellular distribution of actin filaments and cortactin was examined by immunofluorescence. Cells were seeded on Matrigel (1 mg/ml)-coated coverslips in 24-well plates. After 24 h, the growth medium was replaced with the fresh one, containing previously indicated concentrations of inhibitors. Next, the cells were fixed with 4% formaldehyde and permeabilized with 0.1% Triton X-100 in PBS. Coverslips were blocked with 1% bovine serum albumin in PBS. Anti-cortactin antibodies, followed by Alexa Fluor 488–conjugated anti-rabbit secondary antibodies, were applied to visualize this protein. Actin filaments were stained with Alexa Fluor 568–labeled phalloidin and cell nuclei with Hoechst 33342. Then, coverslips were mounted with Dako fluorescent mounting medium. For each condition, cells were imaged (Zeiss LSM 510 confocal laser scanning microscope and ZEN software were used) in three independent experiments, and representative cells are shown. Quantitative analysis of the number of invadopodia per nuclei was performed using ImageJ software (Schneider et al., 2012). Only invadopodia-positive for F-actin and cortactin were scored, and at least 30 cells were analyzed per condition.

### Western Blot Analysis

Twenty four hours after cell seeding, the medium was replaced with the fresh one, and cells were incubated with previously indicated concentrations of inhibitors for 4 h. Cell lysates were harvested by addition of CB buffer (10 mM Tris, pH 7.4, 100 mM NaCl, 1 mM EDTA, 1 mM EGTA, 1 mM NaF, 20 mM Na_4_P_2_O_7_, 2 mM Na_3_VO_4_, 1% Triton X-100, 10% glycerol, 0.1% SDS, 0.5% deoxycholate) supplemented with protease and phosphatase inhibitors cocktails (Sigma). Protein concentration was determined with standard Bradford procedure (Sigma) (Bradford, 1976). Samples of an identical amount of protein (20 μg) were separated by 10% polyacrylamide gel electrophoresis in the presence of sodium dodecylsulfate (SDS-PAGE) according to Laemmli (Laemmli, 1970) and then transferred to nitrocellulose sheets, according to Towbin et al. (Towbin et al., 1979). Antibodies to Src, pSrc, GAPDH, as well as goat anti-mouse antibodies conjugated with horseradish peroxidase (Cell Signaling Technologies) were applied according to the manufacturer’s protocols. Immunoblots were developed using the Clarity Western ECL Substrate (Bio-Rad), scanned with ChemiDoc (Bio-Rad) and analyzed with ImageLab Software (ver. 6.0, Bio-Rad). At least three independent experiments were conducted.

### Isolation of Melanoma Cells From Patients’ Biopsies

Melanoma samples from primary and metastatic tumors from seven biopsies derived from skin melanoma patients were obtained during surgical interventions in Lower Silesian Cancer Center, Wroclaw, Poland. Histopathological analyses were carried out to confirm the melanocytic characteristics of tumor specimens. The study was permitted on the 16.09.2015 by the Ethical Committee of the Regional Specialist Hospital in Wroclaw, Research and Development Centre, Wroclaw, Poland (decision number: KB/21/2015). The experiments were executed with the understanding and written consent of all patients involved in the study. The study methodologies conformed to the standards set by the Declaration of Helsinki.

Melanoma cells were isolated as previously described (Sztiller-Sikorska et al., 2012). Briefly, tumor fragments were minced using sterile scalpels and then incubated in RPMI 1640 medium (Sigma) supplemented with 0.01% DNase I (Sigma) and 0.5% collagenase IV (Invitrogen) for 2–3 h at 37°C. After centrifugation, isolated cells were seeded in complete medium (RPMI 1640 with 20% FBS) on Matrigel (1 mg/ml)-coated coverslips. Twenty four hours later, the medium was changed to serum-free medium consisting of DMEM/F12 (Gibco), B-27 supplement (Gibco), 10 ng/ml basic fibroblast growth factor (PreproTech), insulin (10 mg/ml) (Sigma), heparin (1 ng/ml) (Sigma), EGF (20 ng/ml), and antibiotics (100 U/ml penicillin, 100 μg/ml streptomycin, 25 µg/ml amphotericin B). After 24 h, the medium was replaced by the new one supplemented with previously indicated concentrations of inhibitors and growth factors for the next 24 h, and immunofluorescence staining was performed.

### Fluorescent-Gelatin Degradation Assay

The experiment was conducted according to the procedure described by Artym (Artym et al., 2006). Poly-L-lysine-coated coverslips were washed with PBS and fixed with 0.5% glutaraldehyde for 15 min. Then, the coverslips were inverted on a 30μl drop of gelatin conjugated with FITC (fluorescein) and incubated for 10 min. After washing with PBS, the residual reactive groups were quenched with 5 mg/ml sodium borohydride for 1 min and washed with PBS. Cells were plated in 24-well plates containing a coverslip coated with fluorescent gelatin matrix and incubated at 37°C in the presence of inhibitors. After 16 h, cells were fixed with 4% formaldehyde, permeabilized with 0.1% Triton X-100, and labeled for filamentous actin with Alexa Fluor 568 phalloidin. Confocal images were acquired using the Olympus FV500 confocal laser scanning microscope and FluoView software (Olympus). Sites of degraded matrix were visible as dark areas (spots) in the bright green fluorescent gelatin matrix. The area of gelatin digestion and number of digesting cells were calculated for 20 cells per condition using ImageJ software (Schneider et al., 2012). Experiment was performed in triplicate.

### Gelatin Zymography

The activity of secreted gelatinases — MMP2 (matrix metalloproteinase 2) and MMP9 (matrix metalloproteinase 9) — was determined using cell-conditioned media. Cells were seeded on 60-mm Petri dishes in complete medium. After 24 h, the culture medium was replaced by serum-free medium supplemented with previously indicated concentrations of inhibitors and growth factors. After 48 h of incubation at 37°C, the medium was collected and concentrated about 20 times using Amicon^®^ Ultra-4 Centrifugal Filters (Merck Millipore). Then, after determination of protein concentration by Bradford method (Bradford, 1976), cell-conditioned media were analyzed on SDS-polyacrylamide gels containing 1 mg/ml gelatin. The gels were stained with Coomassie Brilliant Blue R-250 (Sigma), and MMPs activity was detected as transparent bands on the blue background. Experiment was performed in triplicate.

### Statistical Analysis

All data are given as means ± standard deviations (SD), and their significance was determined with GraphPad Prism 7 software using one-way ANOVA followed by Bonferroni test (migration and invasion assays) or Kruskal–Wallis method followed by Dunn’s *post hoc* test (number of invadopodia, number of digesting cells and digestion area).

## Results

### Effects of EGFR and MET Inhibitors on the Migration and Invasion Abilities of Melanoma Cells

All experiments were conducted on three melanoma cell lines—one isolated from primary tumor (A375), and two derived from lymph node metastasis (Hs294T and WM9). All of them exhibit EGFR and MET expression [as described earlier by our group (Dratkiewicz et al., 2018)]. Our previous results showed that combination therapy, composed of EGFR (gefitinib or lapatinib) and MET (foretinib) inhibitors, was much more effective against melanoma cells in a comparison to a monotherapy (Dratkiewicz et al., 2018). The inhibitors decreased the viability of cells in two-dimensional conditions in a significant way; however, some of the cells were still able to survive (Dratkiewicz et al., 2018). We assume that, even if there is a population of cells that is able to survive the inhibitors treatment but does not exhibit the capability to metastasize, it will be a huge benefit for the patient. Therefore, in the next step of our research, we verified the effect of previously established concentrations of inhibitors on migration capacities of examined melanoma cells. Throughout all experiments, cells were treated with 5 nM EGF and 30 ng/ml HGF to mimic conditions present in the melanoma microenvironment. Melanoma cells often overproduce EGF, which in turn by the autocrine stimulation positively influences their growth and rate of metastasis. Moreover, activated fibroblasts present in the cancer cells niche secrete HGF, which is also described as mitogenic factor for melanocytes and can increase their invasion (Li et al., 2003; Elias et al., 2010; Makowiecka et al., 2016).

**Figure 1:**
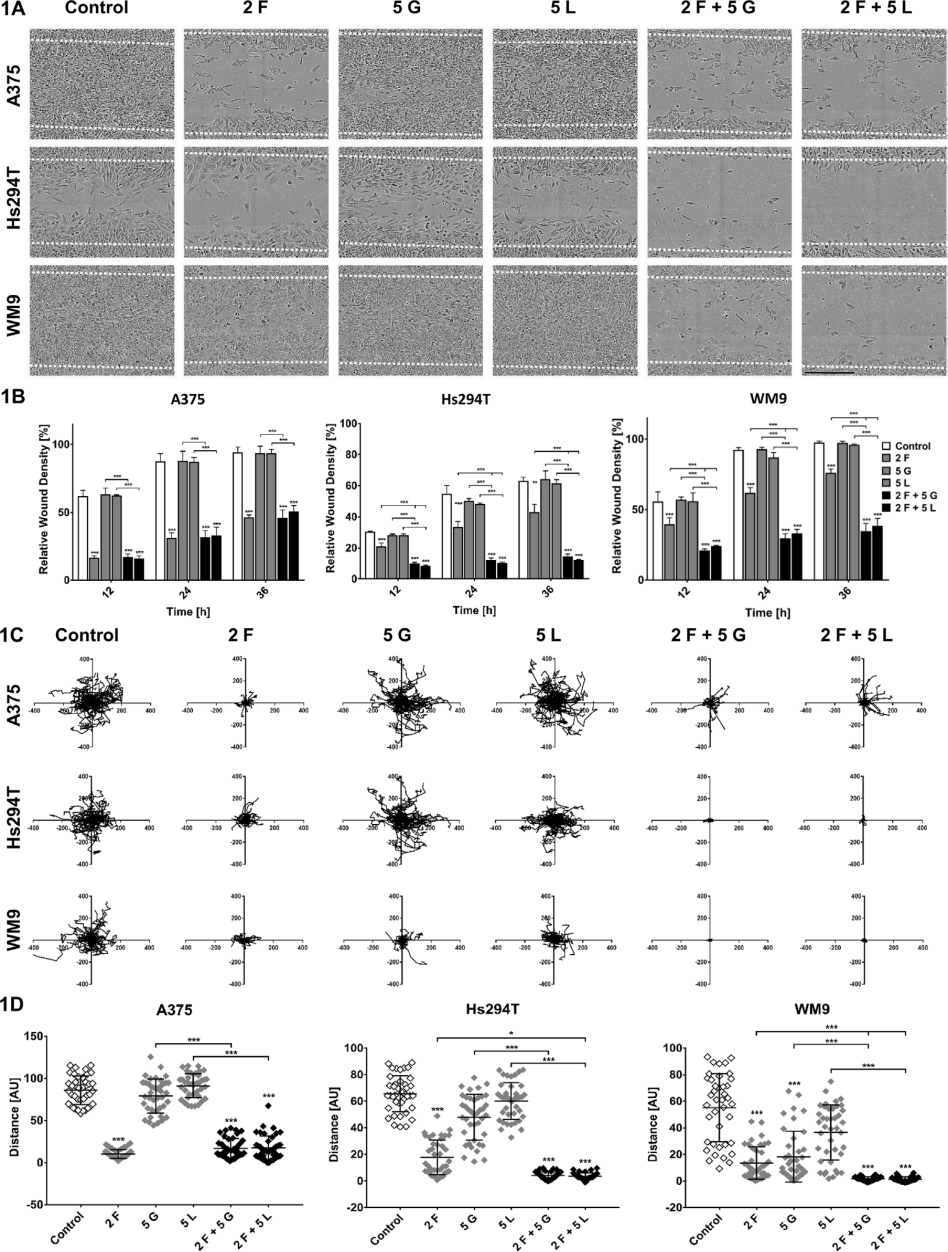
Migration capacities of melanoma cells treated with inhibitors. A375, Hs294T, and WM9 cells were seeded on a thin layer of Matrigel and then incubated with foretinib [F], gefitinib [G], and lapatinib [L] or their combinations at the indicated concentrations (µM) for 36 h (A, B) or 48 h (C, D). **(A)** Exemplary pictures illustrating wound closure after 36 h. **(B)** Relative wound density was continuously measured and quantified based on pictures captured with an IncuCyte® Scratch Wound Cell Migration Software Module. **(C)** Cell trajectories and **(D)** migration distances were analyzed during 48 h of inhibitors treatment using IncuCyte® Live-Cell Analysis System and ImageJ software. Results are expressed as the mean ± SD and are based on at least three independent experiments. Asterisks indicate differences between control and treated cells or between cells treated with different drugs. The significance level was set at p ≤ 0.05 (*) and p ≤ 0.001 (***).

In the first step, migration imitating movement of cells in two-dimensional (2D) conditions e.g., on surface of basement membrane was evaluated using directional migration scratch assay. We noticed that foretinib, as well as pairs of inhibitors, decreased the ability of cells to close the wound (**Figure 1A**). The mixes of drugs worked more effectively than the monotherapy in the case of metastasis-derived cell lines—WM9 and Hs294T, while in A375 cells, their effect was similar to treatment with foretinib alone (**Figure 1B**). Additionally, we conducted a spontaneous migration assay, where cells were seeded sparsely, and there was no attractant causing directional migration. Results of this assay were analogous to these obtained for directional migration (**Figures 1C, D**). Distances covered by cells incubated with foretinib or its combinations with EGFR inhibitors were much shorter than these reached by control cells and cells treated with gefitinib or lapatinib alone (**Figures 1C, D**). Cells treated with mixes, especially WM9 and Hs294T, covered shorter distances compared to cells incubated only with foretinib (**Figure 1D**).

**Figure 2:**
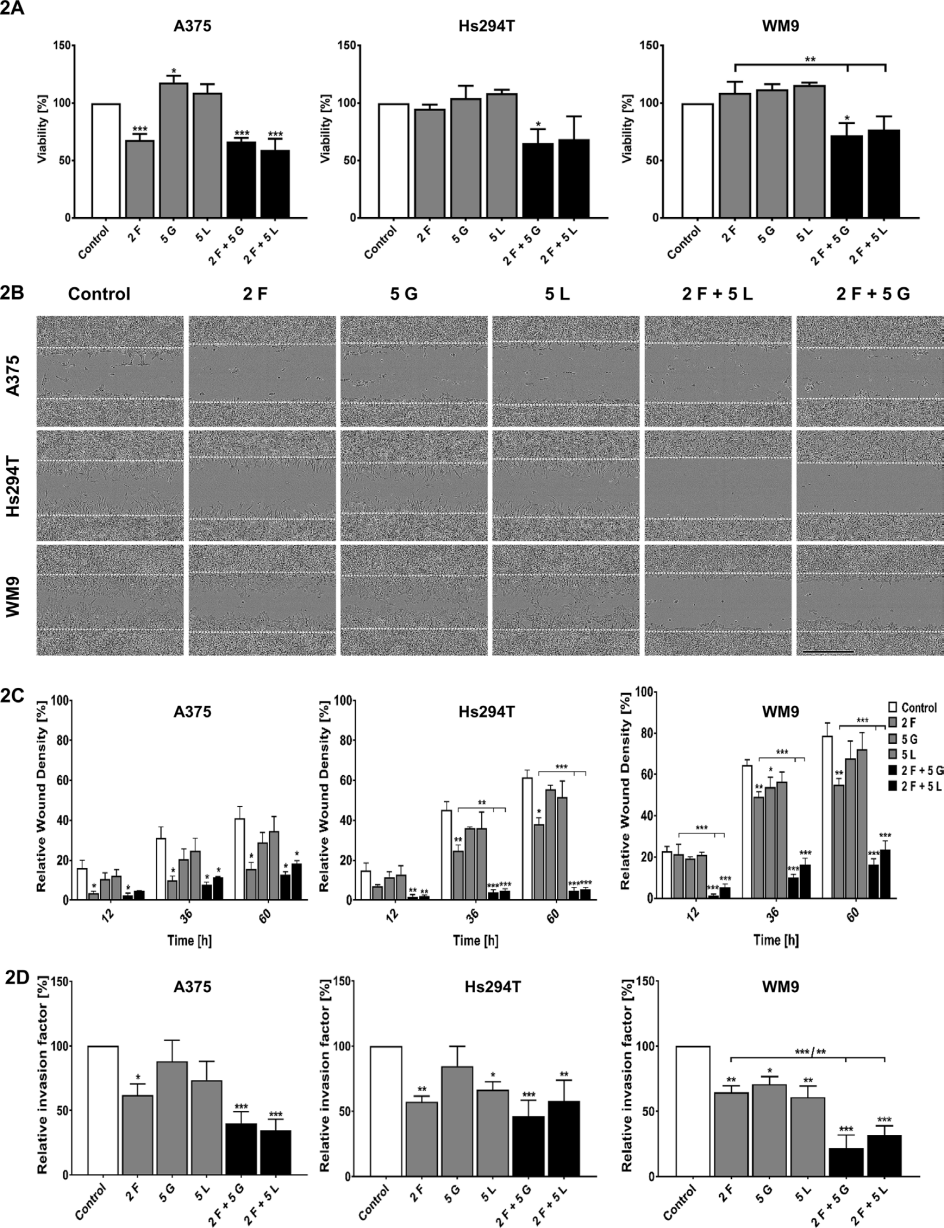
Viability in 3D conditions and invasion capacities of melanoma cells treated with inhibitors. **(A)** Viability of melanoma cells present in three-dimensional conditions treated for 24 h with indicated concentrations of foretinib [F], lapatinib [L], and gefitinib [G] independently, or in combinations, was compared to viability of control cells. Results are expressed as the mean (% of control) ± SD of three independent experiments. Asterisks above the bars express significance vs. control unless indicated otherwise. p ≤ 0.05 (*), p ≤ 0.01 (**), p ≤ 0.001 (***). **(B,C)** Cells were embedded between two layers of Matrigel and then treated with foretinib [F], gefitinib [G], and lapatinib [L] or their combination at the indicated concentrations (µM) for 60 h. **(B)** Pictures illustrating wound closure. **(C)** Relative wound density calculated based on pictures with an IncuCyte® Scratch Wound Cell Migration Software Module. **(D)** The invasion assay conducted on Transwell filters coated with Matrigel for 24 h. Relative invasion capacity was calculated versus control cells, where number of invading cells is set as 100%. Results are expressed as the mean ± SD and are based on at least three independent experiments. Asterisks indicate conditions statistically different from control cells or between particular treatment conditions. The significance level was set at p ≤ 0.05 (*), p ≤ 0.01 (**), and p ≤ 0.001 (***).

In order to evaluate the efficiency of used drugs in conditions imitating tumor environment, we conducted XTT assay, assessing cells viability in 3D (three-dimensional) conditions, where cells were embedded between two layers of Matrigel. Obtained results indicate that 50–70% of cells (depending on the cell line) are able to survive treatment with drugs mixtures in these experimental settings (**Figure 2A**). Next, we performed invasion assays to determine if inhibitors are able to influence in the same extent the cells present in 2D and 3D conditions (**Figures 2B–D**). First, in a 3D wound-healing invasion assay, where a confluent cell population embedded between two Matrigel layers invaded a cell-free area, we observed that examined cells migrated much slower to close the wound; they were much more elongated and less flattened than in two-dimensional conditions (**Figure 2B**). The relative wound density was lower for the cells treated with inhibitors in comparison to the control cells (**Figure 2C**). Furthermore, invasion was significantly decreased for WM9 and Hs294T cells treated with a combination of foretinib with gefitinib or lapatinib than for cells incubated with single agents. However, this effect was less visible in the case of A375 cells. Then, Boyden chamber invasion assays were performed, in which cells invaded though a Matrigel present on top of the membrane. A significant decrease in the invasion capacity was observed in the case of cells treated with foretinib or its mix with gefitinib or lapatinib for all tested cell lines in comparison to control cells (**Figure 2D**). In A375 and WM9 cells, the effect on invasion was stronger in the case of cells treated with the combination of EGFR and MET inhibitors than for single agents (**Figure 2D**). This occurrence was less evident for Hs294T cells.

Mixtures of inhibitors reduced the viability of cells in 2D by 30–60% (Dratkiewicz et al., 2018) and in 3D environment by 30–50% (**Figure 2A**), while according to obtained results, their ability to migrate and invade was decreased by 80–90% (**Figures 1B** and **2B**). These data suggest that used combinations of inhibitors are able to block invasive abilities even of these melanoma cells which evaded apoptosis under drugs treatment.

### Effect of Inhibitors on Invadopodia Formation

**Figure 3:**
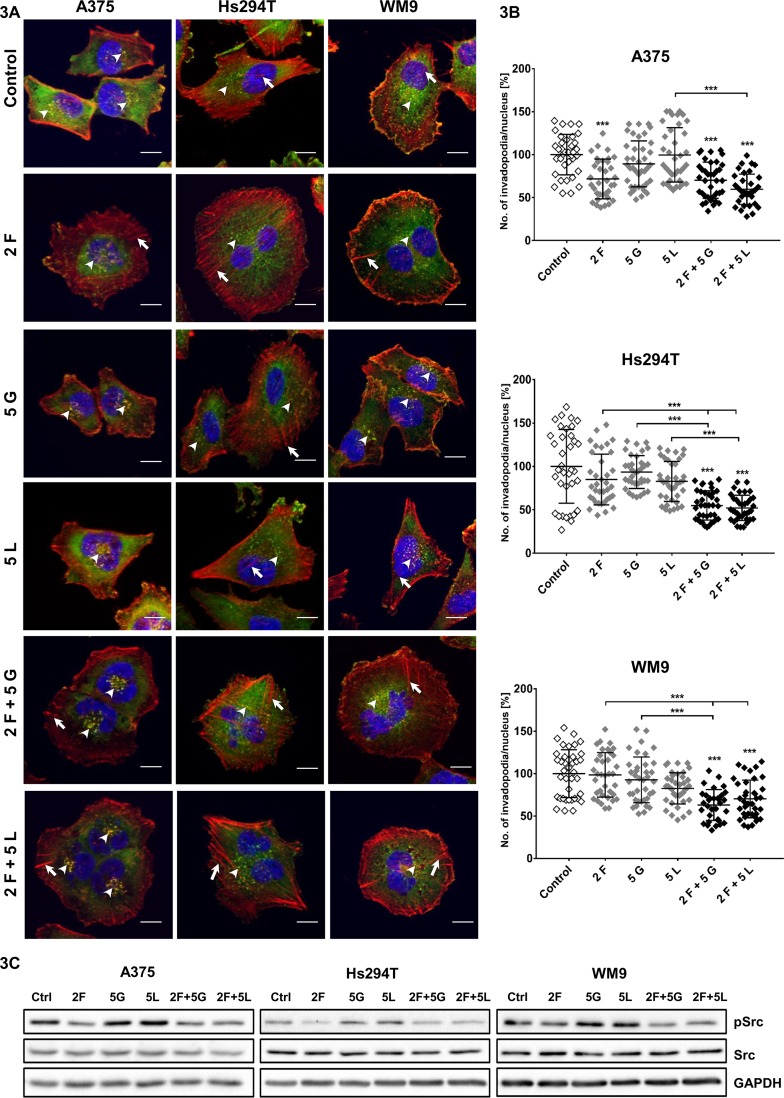
Influence of inhibitors on invadopodia formation in examined melanoma cell lines. **(A)** Representative pictures of A375, Hs294T, and WM9 cells (control or treated for 24 h with indicated concentrations (µM) of foretinib [F], lapatinib [L], and gefitinib [G] independently or in combinations) seeded on Matrigel-coated coverslips stained for F-actin (red), cortactin (green), and cell nuclei (blue). Arrowheads indicate invadopodia, while short arrows mark stress fibers. Scale bar—10 µm. **(B)** Average number of invadopodia per cell nucleus in control and inhibitor-treated cells were calculated using ImageJ software. Invadopodia from 30 cells from three independent experiments were counted. Asterisks indicate conditions statistically different from control cells or differences between inhibitors-treated cells. The significance level was set at p ≤ 0.001 (***). **(C)** Effect of inhibitors treatment on activity of Src kinase. Cells were incubated with indicated concentrations of drugs independently or in combinations for 4 h. Membranes were probed with specific antibodies against total and phosphorylated forms of Src as well as GAPDH and are representative for at least three independent experiments.

Due to the fact that actin cytoskeleton is inseparably linked to the process of cell migration, we analyzed its organization in examined cells. In this study, our attention was focused especially on invadopodia — actin-rich adhesive structures with proteolytic activity, which are often formed by mesenchymally migrating cells. Cancer cells form these protrusions to digest the elements of the ECM and to create paths used later to invade through the tissues (Gimona et al., 2008). Our earlier studies shown that tested melanoma cells are able to form invadopodia (Makowiecka et al., 2016). In drug-treated cells, the filamentous actin (F-actin) organization and cortactin (a marker of invadopodia) were visualized (**Figure 3A**). Invadopodia were visible as dots in the cell nuclei proximity, where F-actin and cortactin colocalized (**Figure 3A**, arrowheads).

As we previously observed, the most significant changes were present in cytoskeleton organization of melanoma cells after administration of foretinib alone and paired with gefitinib or lapatinib (Dratkiewicz et al., 2018). These cells were larger and more spread and formed more pronounced actin stress fibers (short arrows) compared to control- and gefitinib/lapatinib–treated cells. EGFR inhibitors did not affect cell morphology or actin cytoskeleton organization significantly. We also noticed that treatment with foretinib used as a monotherapy or in combination with gefitinib or lapatinib led to the appearance of cells containing multiple or larger nuclei in comparison to non-treated cells (**Figure 3A**). This is in line with the effect of foretinib (or combination of this drug with EGFR inhibitor) that was observed by us earlier during the cell cycle analysis (Dratkiewicz et al., 2018). We assume that cells could undergo nuclei division, but cytokinesis did not occur, which resulted in emergence of multinucleated cells. This is the reason why we calculated number of invadopodia per cell nuclei, instead of the number of these structures per cell (**Figure 3B**). Cells treated with inhibitors are still able to form invadopodia; however, upon quantification of these structures, we noticed a reduction in their number in cells treated with the inhibitors mixtures compared to control cells. This effect was not visible when inhibitors were used only as a monotherapy. Additionally, using Western Blot analysis, we verified the level of phosphorylated Src kinase in tested cells. In its active form, this protein constitutes the main signaling kinase stimulating invadopodia formation and activity (Mader et al., 2011; Burger et al., 2014; Kolli-Bouhafs et al., 2014). We noticed that its level was reduced in cells treated with foretinib and mixtures of inhibitors (**Figure 3C**). Presented results suggest the existence of negative feedback of inhibitors on invadopodia formation or their stability.

### Influence of Inhibitors on Melanoma Cells Isolated From Patients’ Biopsies

**Figure 4:**
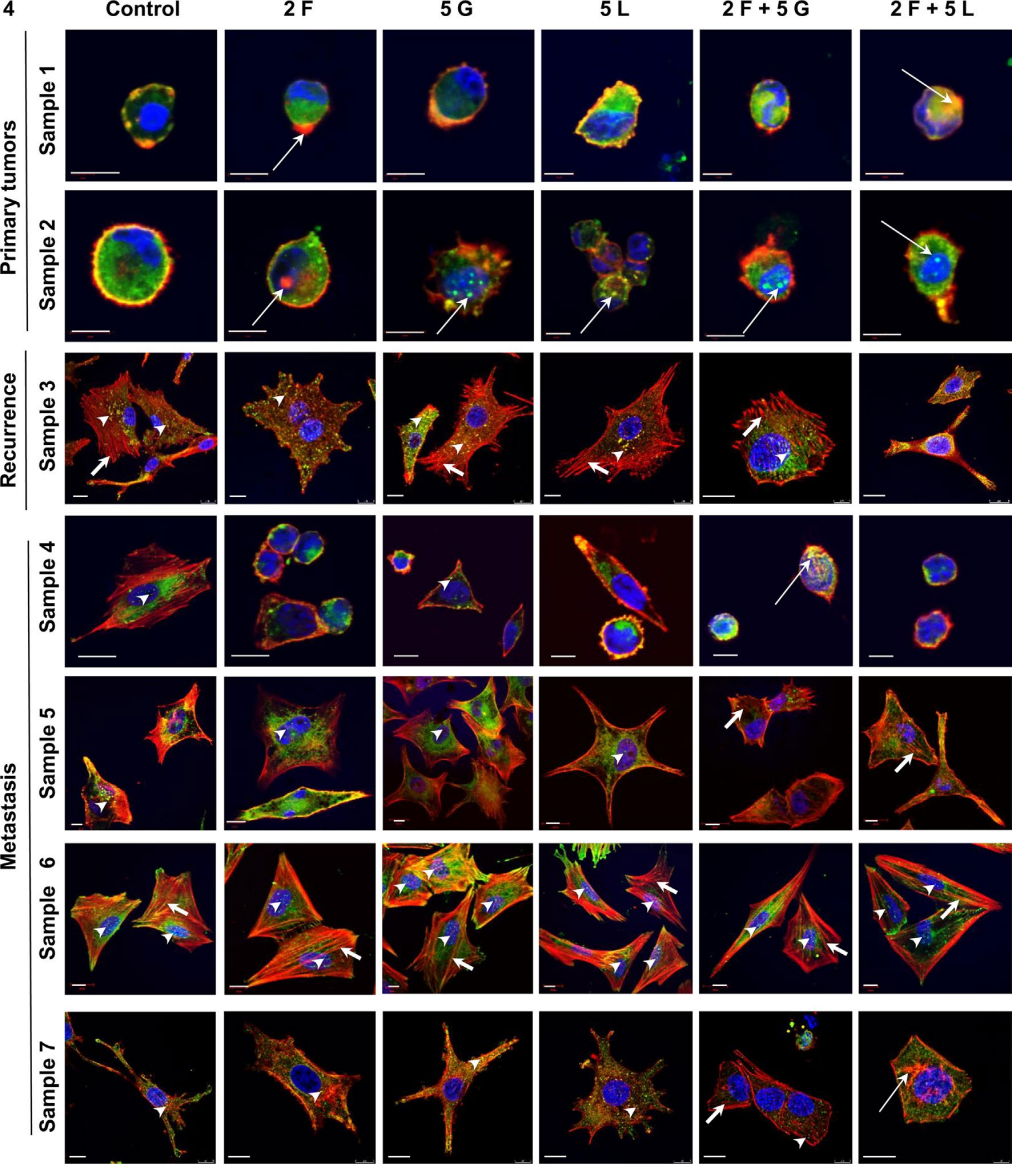
Influence of inhibitors on actin cytoskeleton organization and invadopodia formation in melanoma cells obtained from patients. Cells were isolated from patients’ biopsies derived from primary tumors, recurrence or metastasis, seeded on Matrigel-coated coverslips and treated for 24 h with selected concentrations (µM) of inhibitors: foretinib [F], lapatinib [L], and gefitinib [G] independently or in combinations. Cells were labeled to visualize F-actin (red), cortactin (green), and cell nuclei (blue). Arrowheads indicate invadopodia, short arrows point to stress fibers, and long arrows point to actin aggregates. Scale bar—10 µm.

To relate results obtained using cell lines to clinical data, we isolated melanoma cells from patients’ biopsies (identified by Melan-A staining) and then treated them with foretinib, gefitinib, and lapatinib separately or in combinations. After staining analogously as it was done in the case of cell lines, cells were analyzed using confocal microscopy (**Figure 4**). We noticed the appearance of alterations in cytoskeleton organization and in response to inhibitors treatment between cells which originated from primary tumor, recurrence, and metastasis. Cells derived from primary tumors exhibited predominantly rounded morphology. They did not present well organized actin cytoskeleton and did not form invadopodia. In contrast, cells originating from the recurrence and metastasis were more spread with well-organized cytoskeleton, and migratory protrusions like invadopodia (marked by arrowheads), lamellipodia, or filopodia were clearly visible in all examined cells in control conditions. In part of tested cells, like in sample 4, invadopodia disappeared after incubation with foretinib and lapatinib alone, or following combination therapies. This patient has not been subjected to systemic treatment until the time of biopsy, which can be connected to sensitivity of cells to used drugs. In the case of cells derived from patients 3, 5, 6, and 7, they reacted only partially to the use of inhibitors — mostly after treatment with their mixes. The resistance to applied agents in part of the samples (5 and 6) can be related to the fact that patients, from whom the biopsies were isolated, were earlier treated with radiotherapy or immunotherapy. Cells from samples 3 (recurrence) and 7 (metastasis) were isolated from the same patient, who has not been previously treated with chemo- or radiotherapy but is quite resistant to used inhibitors — invadopodia present in these cells disappeared only after application of combination of foretinib and lapatinib. Additionally, we observed rearrangements in actin cytoskeleton organization after incubation with inhibitors — more stress fibers (short arrows) or actin aggregates (long arrows) were noted. Moreover, treatment with combination of inhibitors resulted in changes of cells shape — they became spherical (sample 4) or elongated and branched (samples 3 and 5) (**Figure 4**).

### EGFR and MET Inhibitors Affect Proteolytic Activity of Examined Melanoma Cells

**Figure 5:**
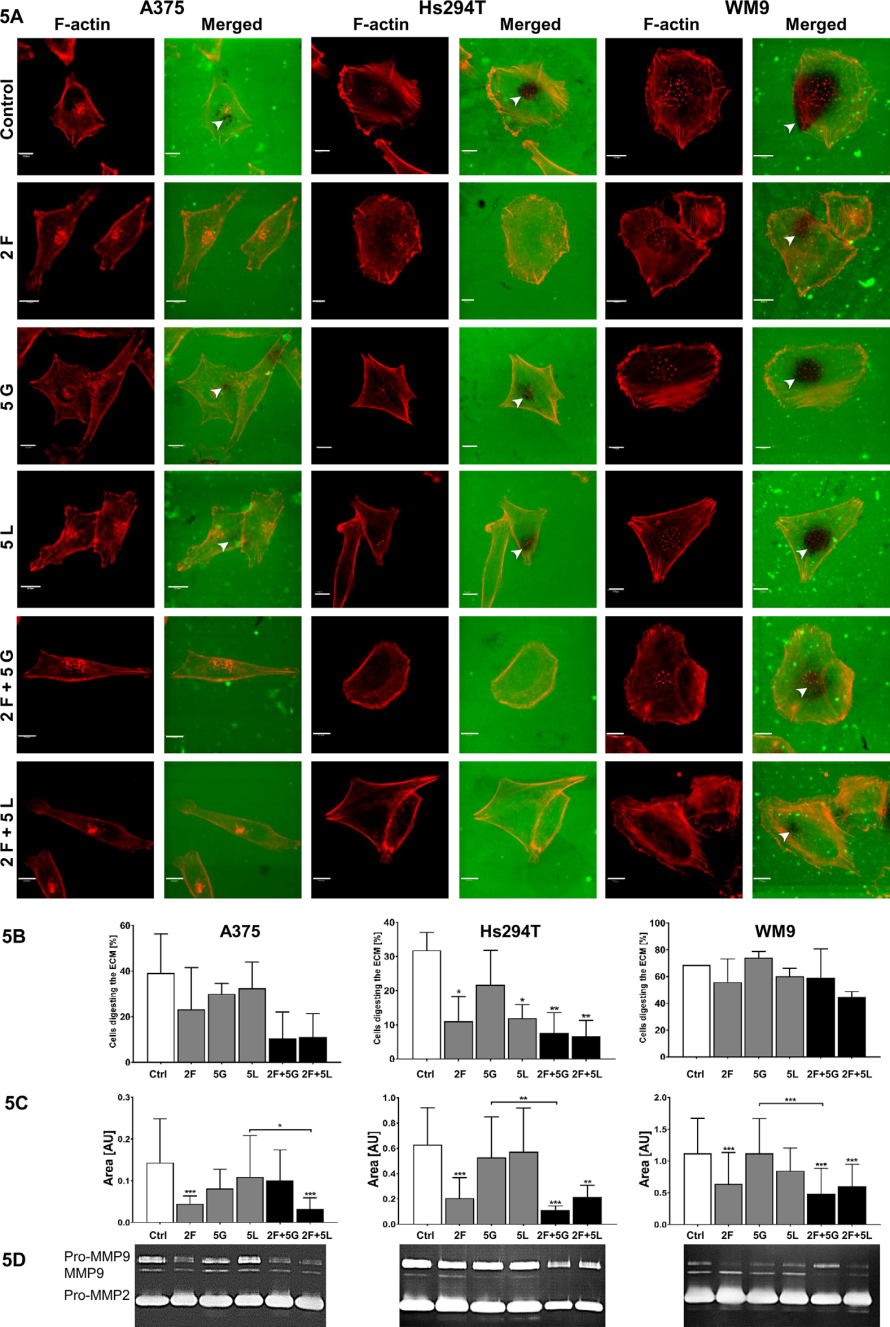
Influence of inhibitors on proteolytic activity of melanoma cells. Analysis of effect of foretinib [F], lapatinib [L], and gefitinib [G] used in selected concentrations (µM) independently or in combinations on melanoma cell lines’ proteolytic activity. **(A)** Cells were seeded onto coverslips coated with FITC-conjugated gelatin (green) and incubated for 16 h with inhibitors. Then, the cells were fixed and stained with Alexa Fluor 568 phalloidin (red) to visualize F-actin. Gelatin degradation is visualized as the dark areas on the fluorescently-labeled gelatin background. Digested areas are indicated with white arrowheads. Scale bar—10 µm. **(B)** Number of cells digesting gelatin as well as **(C)** digestion area calculated using ImageJ software. Asterisks indicate differences between control and treated cells or between cells treated with different drugs. The significance level was set at p ≤ 0.05 (*), p ≤ 0.01 (**), and p ≤ 0.001 (***). **(D)** MMP2 and MMP9 gelatinase activity within media collected from control- and inhibitor-treated cells detected by gelatin zymography.

Since the enzymes digesting ECM are involved in mesenchymal type of movement, in the last step of our research, we determined the proteolytic activity of tested cells after treatment with inhibitors. We performed gelatin-FITC degradation assay, in which sites of gelatin digestion appeared as black spots on a fluorescent background. Additionally, to visualize cell shape and localization of invadopodia, we stained F-actin using phalloidin conjugated with Alexa Fluor 568. Control cells representing all tested lines were able to digest fluorescently-labeled gelatin through secretion of proteolytic enzymes, mainly by invadopodia (**Figure 5A**). Cells treated with foretinib degraded the matrix less distinctly, while cells incubated with inhibitors’ combinations lost the ability to digest gelatin completely or almost completely. We quantified obtained results and noticed that after administration of inhibitors number of cells, which were able to digest ECM, was decreased (results are statistically significant only for Hs294T cells) (**Figure 5B**). The area of digested gelatin was also smaller in comparison to control cells, especially after use of foretinib and combination therapies (**Figure 5C**). Unfortunately, due to the large variety of digestive patterns exhibited by examined cell lines, obtained results are characterized by large standard deviations, which makes differences between cells treated with single drugs and mixtures statistically insignificant. Additionally, we performed gelatin zymography (this method allows to detect active, secreted gelatinases) and noticed that, particularly in cells incubated with pairs of inhibitors, proteolytic activity of MMP9 was lower than in control cells and cells incubated with single agents (**Figure 5D**).

Acquired results suggest that simultaneous blocking of signaling pathways connected to EGFR and MET receptors effectively reduces capability of cells to digest elements of extracellular matrix by limited secretion of matrix metalloproteases and may consequently decrease their ability to invade tissues in a protease-dependent manner.

## Discussion

It is widely known that EGFR and MET stimulate invasion of cancer cells. Activated ErbB receptors (including EGFR) modulate Rho GTPases activity, which leads to actin polymerization and microfilaments reorganization, which is mandatory for cell migration (Feigin and Muthuswamya, 2009; Appert-Collin et al., 2015). Moreover, during the mesenchymal mode of invasion, the ECM undergoes proteolysis, thus resulting in the appearance of matrikines — short peptides arising from fragmentation of matrix proteins. They restrict the influence of EGFR downstream signaling solely to the perimembrane area, which is mostly involved in cell migration, therefore strengthening the effect of EGFR signaling on cancer cell motility (Iyer et al., 2008). MET is also engaged in cell invasion process. Upon phosphorylation of MET, it recruits Gab1 and Gab2, which then activate Shp2, Ras, and ERK/MAPK, as well as Ras–Rac1/Cdc42–PAK and Crk–C3G—Rap1 (Birchmeier et al., 2003; Karamouzis et al., 2009). These signaling pathways promote tumor metastasis changing the expression or activation of extracellular matrix proteases (such as MMPs), as well as cytoskeletal (cadherins, Arp2/3, N-WASP) and cell adhesion molecules (paxillin, integrins, and focal adhesion kinase) (Birchmeier et al., 2003; Cao et al., 2015; Demkova and Kucerova, 2018). Simultaneous treatment with HGF and EGF synergistically increased invasion of mammary epithelial cells. Moreover, EGF, but not HGF treatment, resulted in the increase of MET — at the mRNA and protein level (Accornero et al., 2010). Furthermore, Carpenter et al. indicated that interaction between EGFR, MET, and integrin α6β4 enhances the pancreatic cancer cell motility. Integrin α6β4 signaling increases transcription of EGFR ligands as well as *MMP1* and *EGFR* genes. After stimulation with HGF, MET and integrin α6β4 cooperate to activate MMP1, which leads to activation of EGFR and results in increased cell migration and invasion (Carpenter et al., 2015). Bonine-Summers et al. also reported that HGF-induced EGFR activation enhanced MET signaling, which raised cell proliferation and invasion, while inhibition of EGFR using gefitinib blocked the HGF-mediated cellular responses of breast carcinoma cells (Bonine-Summers et al., 2007). Engelman et al. demonstrated that MET activates ErbB3 signaling in tumor cells, and *MET* gene amplification induces the appearance of cells resistant to gefitinib (Engelman et al., 2007). Abovementioned results confirm the cooperation of these growth factors and their receptors and suggest that combination of EGFR and MET inhibitors prevents the crosstalk between these receptors, which may result in reduction of cell migration and invasion.

The majority of studies focused only on verification if used EGFR inhibitors combined with MET inhibitors synergistically affect cell viability (Mueller et al., 2010; Liu et al., 2011) or tumor growth (Linklater et al., 2016). Previously, we have also showed that pairs of inhibitors directed against EGFR (gefitinib, lapatinib), and MET (foretinib) are able to effectively decrease viability and proliferation, and induce apoptosis in examined melanoma cells. Moreover, after inhibitors' treatment cells were often multinucleated and exhibited massive enrichment in the G2/M cell cycle phase. We also noticed that administration of inhibitors — pairs of foretinib with gefitinib or lapatinib — induced changes in actin cytoskeleton organization (Dratkiewicz et al., 2018). Rearrangements of actin are the basis of cell movement and thus metastasis, which provoked us to continue our research. Here, we evaluated the effect of selected pairs of inhibitors on invasion of melanoma cells. To better mimic the microenvironment of migrating cells, we investigated melanoma cell motility both in 2D, reflecting the migration on the surface of basement membrane, and in 3D conditions, imitating invasion through the surrounding tissue. This work for the first time shows that invasive abilities of melanoma cells are decreased after application of pairs of EGFR and MET inhibitors.

Our results indicate that both directed and spontaneous types of migration (2D conditions) of melanoma cells are inhibited by foretinib, and its combinations with gefitinib or lapatinib. The effect of treatment with pairs of inhibitors is stronger for cell lines derived from metastases (WM9, Hs294T) compared to primary (A375) tumors, both in terms of wound closure abilities and distances covered by cells. Similar data were acquired in 3D conditions, where cells were embedded between two layers of Matrigel. After addition of foretinib and its mixtures, cells invading through Matrigel-coated Transwell filters also presented decreased abilities to cross the barrier; however, only in the case of WM9 cells, the difference in response to this treatment was visible. Analogous phenomenon was noticed by Lee and co-workers who showed that ME22S (a novel EGFR/MET bispecific antibody) significantly inhibited HGF-stimulated migration and invasion of laryngeal carcinoma cells (Lee et al., 2016). Xu et al. also demonstrated that combination of EGFR and MET inhibitors in head-and-neck carcinoma cells decreased the rate of wound closure and invasion of cells (Xu et al., 2011).

One could speculate that decreased cell migration and invasion are an effect of lowered cell viability. However, only small percentage of cells undergoes apoptosis under the influence of used combination of drugs (4–40%, depending on the cell line, see Dratkiewicz et al., 2018). The surviving cells are still proliferating and are able to migrate. Moreover, when we tested viability of cells in 3D conditions, we noticed that it was lowered only by 30–50% at the used drug concentrations, while cell migration and invasion were significantly impaired. Therefore, we would like to emphasize that the aim of anti-cancer therapies is not only the elimination of cancer cells but also the prevention of metastases. Even if the drug is not able to kill the cells but blocks their spreading, it will pose a great benefit for the patient.

To determine the changes in actin cytoskeleton related to decreased cell motility upon treatment with inhibitors, cells were stained for filamentous actin and cortactin. We previously showed that EGF and HGF stimulate invadopodia formation and extracellular matrix degradation, which correlates with higher invasive abilities of melanoma cells (Makowiecka et al., 2016). Here, we noticed the appearance of more pronounced stress fibers and lowered number of formed invadopodia after addition of foretinib and pairs of inhibitors. Again, there was statistically significant difference in invadopodia amount between metastasis-derived cells treated with foretinib alone compared to mixes. EGFR (AG1478 and cetuximab) and MET (SU11274) inhibitors also induced changes in actin cytoskeleton organization of oral squamous cell carcinoma cells. Moreover, MET inhibitor reduced filopodia and lamellipodia formation, thus diminishing migratory abilities of these cells (Yasui et al., 2017). Fichter et al. also indicated that, upon EGFR inhibition by gefitinib or lapatinib, the number of filopodia and microspikes present in esophageal cells was decreased, with simultaneous induction of focal adhesions and stress fibers formation (Fichter et al., 2014). Additionally, treatment of HGF-stimulated cholangiocarcinoma cells with MET siRNA led to the disappearance of actin-rich protrusions (Leelawat et al., 2006). Based on abovementioned results, we postulate that one of the mechanisms, by which EGFR and MET inhibitors decrease cell migration abilities, is the reduction of protrusive activity of examined cells. This thesis is strengthened by the fact that the level of phosphorylated Src kinase is lowered in cells treated with drug mixtures. It was previously shown that EGF signaling activates Src kinase, which is required for cortactin phosphorylation and actin polymerization at places of invadopodia formation (Mader et al., 2011). Therefore, if we block the activity of the EGFR receptor (and possible crosstalk in downstream signaling with the MET receptor), the Src kinase will remain inactive and will not be able to stimulate actin polymerization, which is necessary for the generation of an invadopodial protrusive force that enables cancer cells to invade through the matrix and metastasize to distant organs.

To further our analysis, samples derived from melanoma suffering patients were examined. This part of the experiments involved several challenges. Firstly, we did not have access to a large amount of clinical material, and secondly, isolation of viable melanoma cells from biopsies was problematic. This may be connected with the fact that these cells lost the microenvironment supporting them *in vivo* after transfer to the *in vitro* environment. Moreover, it is difficult to draw unambiguous conclusions from these results due to the large inter-individual variability between samples. Despite these difficulties, we performed several F-actin and cortactin stainings and noticed the appearance of actin aggregates, more pronounced stress fibers, and reduced invadopodia formation after inhibitors treatment, especially in the case of combined therapy. Additionally, we observed a similar tendency in response to treatment in biopsy-derived cells compared to the cell lines — cells derived from primary tumors reacted in less notable way to application of pairs of inhibitors in comparison to cells derived from recurrence or metastasis. Furthermore, cells isolated from biopsies responded weaker to inhibitors treatment than established cell lines. It can be related to the fact that some patients have been previously exposed to alternative forms of treatment and, therefore, drug resistance might have already occurred.

The ability of cells to degrade the ECM greatly affects cell invasion. For this reason, we also analyzed proteolytic activity of melanoma cells. We noticed that, after treatment with pairs of inhibitors, lower number of cells was able to digest fluorescently- labeled gelatin in comparison to control conditions. Additionally, proteolytic activity of these cells was diminished. Moreover, combinations of inhibitors, and in less extent monotherapy, decreased the amount of secreted MMP2 and MMP9. Zhuo *et al*. indicated that EGF in cooperation with HGF increased secretion of MMP9, while addition of MMP9 inhibitor or an anti-MMP9 neutralizing antibody abolished EGF- and HGF-stimulated cell invasion (Zhou et al., 2007). Our data are also in line with results obtained by Zuo et al. who showed that pharmacologic inhibition of EGFR (using AG1478 compound activity) lowered the level of phosphorylated ERK and AKT, and reduced the production of MMP9 as well as cell migration and invasion (Zuo et al., 2011).

Our results for the first time indicate that combination of EGFR and MET inhibitors decrease melanoma cell migration and invasion. These drugs resulted in changes in cytoskeleton organization of cells derived from cell lines and from patient biopsies observed as reduction of the amount of invadopodia as well as appearance of more distinct stress fibers. We noticed that cell lines were more sensitive to drugs treatment than cells isolated from the biopsies. In both cases, cells derived from metastasis responded to a greater extent to mixtures of inhibitors than cells derived from primary tumor. Proteolytic activity of examined cells was also reduced after usage of foretinib with gefitinib or lapatinib. The direct influence of the drug mixtures on the invasion process is supported by the fact that, upon their administration, the level of phosphorylated Src, the kinase responsible for the formation of active invadopodia, necessary for invasion of mesenchymally migrating cells, is decreased (Bowden et al., 2006; Mader et al., 2011; Kolli-Bouhafs et al., 2014).

## Conclusions

We conclude that dual inhibition of EGFR and MET provides greater response in cancer cells, which is construed as decrease of invasive abilities by reduction of protrusive activity, in comparison to monotherapy that may contribute to development of more efficient anti-melanoma therapy for patients exhibiting overexpression of these growth factor receptors. Investigating the effects of drugs on cell invasion is particularly important, because a part of cells that does not undergo apoptosis under the inhibitors treatment can still migrate and form metastases. Therefore, finding a combination of drugs that could inhibit this process would offer a great benefit for the patient. Abovementioned results are promising; however, we realize that the experimental model which we employed is limited. It does not involve the influence of many components of tumor microenvironment on treatment efficacy. Due to that, we would like to continue our research and determine how, other than through EGF and HGF stimulation, the extracellular conditions affect the response of cells to used drugs.

## Data Availability Statement

All datasets generated for this study are included in the manuscript/supplementary files.

## Ethics Statement

This study was carried out in accordance with the recommendations of the Ethical Committee of the Regional Specialist Hospital in Wroclaw, Research and Development Centre, Wroclaw, Poland with written informed consent from all subjects. All subjects gave written informed consent in accordance with the Declaration of Helsinki. The protocol was approved by the the Ethical Committee of the Regional Specialist Hospital in Wroclaw, Research and Development Centre, Wroclaw, Poland (decision number: KB/21/2015).

## Author Contributions

AS, KP-G and DN contributed conception and design of the study; ED performed the statistical analysis; Experiments were conducted by AS, KP-G, ED, MP, MZ and DN. DN and RM supervised the project. AS and DN wrote the first draft of the manuscript. All authors contributed to manuscript revision, read and approved the submitted version.

## Funding

This research was funded by National Science Centre, Poland (OPUS 8, No.2014/15/B/NZ5/01467), grant, received by Dorota Nowak.

## Conflict of Interest

The authors declare that the research was conducted in the absence of any commercial or financial relationships that could be construed as a potential conflict of interest.
